# Supplementary material for: What Happens in Male Dogs after Treatment with a 4.7 mg Deslorelin Implant? II. Recovery of Testicular Function after Implant Removal
Source: Animals (Basel). 2022 Sep 23;12(19):2545. doi: 10.3390/ani12192545 (PMC9559295; doi:10.3390/ani12192545)
Supplement: Supplementary file 1 [file animals-12-02545-s001.zip › animals-1855716-supplementary.pdf]

Table S1. Results of semen analysis of TG (n=5) during restart of spermatogenesis following implant removal; results are given as median (Q1, Q3).

Time (days)	VOL (mL)	% PM	% VS	% MAS	TSC (x10^6)
49	0.2 (0.1, 2.4)	*	*	*	*
56	5.4 (0.3, 6.0)	*	*	*	*
63	2.0 (1.3, 4.9)	*	*	*	*
70	9.0 (3.9, 10.7)	*	*	*	*
77	6.6 (5.8, 10.3)	*	*	*	*
84	6.0 (5.5, 6.6)	80 (75, 80)	92.0 (88.5, 94.0)	*	409.7 (36.3, 515.6)
91	6.7 (5.4, 7.3)	85 (80, 85)	91.5 (90.5, 92.5)	22.0 (18.0, 52.0)	350.0 (68.9, 360.0)
98	4.9 (3.1, 9.6)	80 (75, 80)	90.5 (89.5, 91.0)	25.0 (18.0, 39.5)	265.7 (240.7, 330.0)
105	6.4 (3.5, 7.0)	75 (75, 80)	89.5 (89.0, 92.5)	22.0 (21.5, 32.5)	190.5 (160.0, 370.3)
112	8.4 (8.2, 10.0)	80 (75, 85)	89.5 (88.5, 92.0)	24.0 (13.5, 26.5)	567.2 (484.0, 910.0)
119	6.6 (5.6, 9.1)	80 (75, 85)	96.0 (95.0, 96.0)	15.0 (10.5, 17.0)	525.0 (342.7, 532.8)
126	8.8 (7.8, 10.1)	85 (80, 85)	94.0 (92.0, 95.5)	12.5 (8.0, 14.0)	465.3 (307.0, 590.6)
133	7.8 (7.0, 10.4)	85 (80, 85)	93.0 (91.5, 96.0)	15.5 (10.5, 17.0)	654.6 (526.6, 684.1)
140	9.7 (7.5, 11.3)	85 (80, 85)	94.5 (93.0, 95.5)	14.5 (14.0, 17.0)	412.5 (411.7, 819.5)
147	8.7 (7.0, 10.3)	80 (75, 85)	93.5 (91.0, 95.0)	9.5 (8.5, 17.5)	365.2 (344.5, 502.0)

* analysis not possible in all dogs due to low ejaculate volume (sperm rich fraction).
